# Differential influences of allometry, phylogeny and environment on the rostral shape diversity of extinct South American notoungulates

**DOI:** 10.1098/rsos.171816

**Published:** 2018-01-31

**Authors:** Helder Gomes Rodrigues, Raphaël Cornette, Julien Clavel, Guillermo Cassini, Bhart-Anjan S. Bhullar, Marcos Fernández-Monescillo, Karen Moreno, Anthony Herrel, Guillaume Billet

**Affiliations:** 1 Centre de Recherche sur la Paléobiodiversité et les Paléoenvironnements (CR2P), UMR CNRS 7207, CP38, Muséum National d'Histoire Naturelle, Univ Paris 6, 8 rue Buffon, 75005 Paris, France; 2 Mécanismes adaptatifs et évolution (MECADEV), UMR 7179, CNRS, Funevol team, Muséum National d'Histoire Naturelle, 55 rue Buffon, Bat. Anatomie Comparée, CP 55, 75005, Paris Cedex 5, France; 3 Institut de Systématique, Evolution, Biodiversité (ISYEB UMR 7205), MNHN, CNRS, UPMC, CP26, Sorbonne Universités, 57 rue Cuvier, 75005 Paris, France; 4 Institut de Biologie de l'Ecole Normale Supérieure (IBENS), CNRS UMR 8197, INSERM U1024, Ecole Normale Supérieure, Paris Sciences et Lettres (PSL), Research University, 75005 Paris, France; 5 División Mastozoología, Museo Argentino de Ciencias Naturales ‘‘Bernardino Rivadavia’’(MACN), Ciudad Autónoma de Buenos Aires, Argentina; 6 Departamento de Ciencias Básicas, Universidad Nacional de Luján (UNLu), Luján, Buenos Aires, Argentina; 7 Consejo Nacional de Investigaciones Científicas y Técnicas (CONICET), Yale University, PO Box 208109, New Haven, CT 06520, USA; 8 Department of Geology and Geophysics, Yale University, PO Box 208109, New Haven, CT 06520, USA; 9 Peabody Museum of Natural History, Yale University, 170 Whitney Avenue, New Haven, CT 06511, USA; 10 Instituto Argentino de Nivología, Glaciología y Ciencias Ambientales (IANIGLA), CCT–CONICET–Mendoza, Avenida Ruiz Leal s/n, Parque Gral, San Martín 5500, Mendoza, Argentina; 11 Instituto de Ciencias de la Tierra, Universidad Austral de Chile, Casilla 567, Valdivia, Chile

**Keywords:** mammals, masticatory apparatus, geometric morphometrics, convergences, adaptation, evolutionary rates

## Abstract

Understanding the mechanisms responsible for phenotypic diversification, and the associated underlying constraints and ecological factors represents a central issue in evolutionary biology. Mammals present a wide variety of sizes and shapes, and are characterized by a high number of morphological convergences that are hypothesized to reflect similar environmental pressures. Extinct South American notoungulates evolved in isolation from northern mammalian faunas in highly disparate environments. They present a wide array of skeletal phenotypes and convergences, such as ever-growing dentition. Here, we focused on the origins of the rostral diversity of notoungulates by quantifying the shape of 26 genera using three-dimensional geometric morphometric analysis. We tested the influence of allometry and phylogeny on rostral shape and evaluated rates of evolutionary change in the different clades. We found strong allometric and phylogenetic signals concerning the rostral shape of notoungulates. Despite convergent forms, we observed a diffuse diversification of rostral shape, with no significant evidence of influence by large-scaled environmental variation. This contrasts with the increase in dental crown height that occurred in four late-diverging families in response to similar environmental pressures. These results illustrate the importance of considering both biological components and evolutionary rates to better understand some aspects of phenotypic diversity.

## 1. Introduction

During their evolutionary history, mammals underwent numerous events of diversification that produced a large variety of shapes, including spectacular examples of morphological convergence (e.g. [1,2]). South American ungulates represent an extraordinary case, which illustrates this shape diversity [3] by encompassing both generalist and specialized taxa, some of which were described by Charles Darwin [4] as being among ‘the strangest animals ever discovered'. In fact, the native South American ungulates have long puzzled palaeontologists, including Simpson [5], for their impressive morphological dualism: ‘on one hand, they are remarkably exotic in comparison with the fossil or recent mammals of any other continent, and on the other they parallel these mammals in many features, now considered largely adaptive or secondary, in a way often amazing’. Among them, notoungulates, which appeared during the Late Palaeocene and became extinct during the Pleistocene–Holocene transition, present the largest diversity of sizes and forms. Despite having recently been placed phylogenetically as a sister-group of perissodactyls (i.e. horses, rhinos) based on molecular evidence (e.g. [6]), they show numerous morphological convergences with other groups of extant mammals, such as rodents, rabbits or hyraxes (e.g. [7,8]).

**Figure 1.**
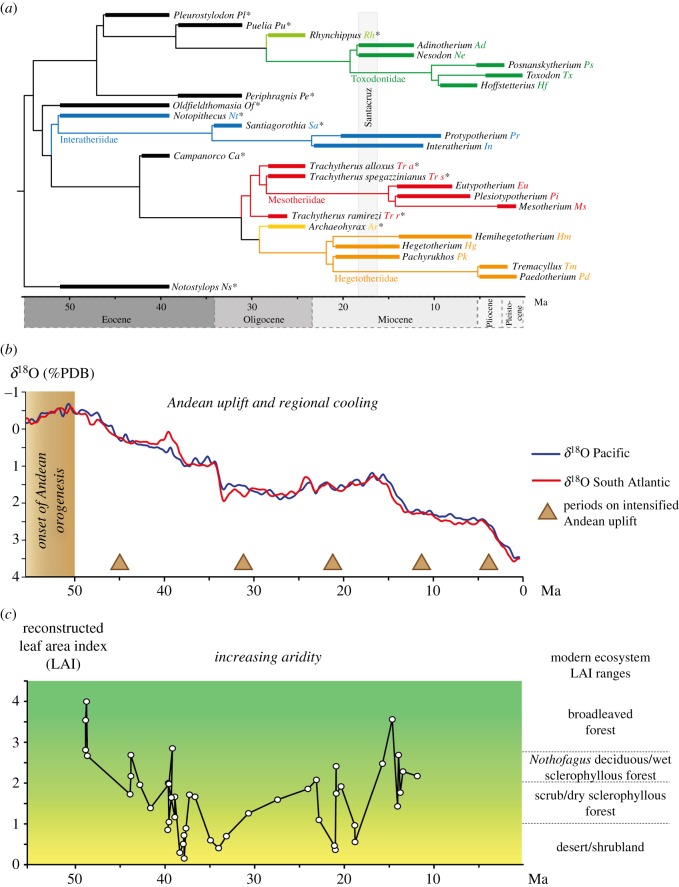
(*a*) Phylogenetic relationships and stratigraphic range of notoungulate taxa. Abbreviations (used in following figures) are indicated after each taxon, with ‘asterisks’ for Palaeogene forms. (*b*) Climatic and geological variations in South America and (*c*) environmental variations in Patagonia during the Cenozoic (modified from [11]).

Late-diverging families of notoungulates (Toxodontidae, Interatheriidae, Hegetotheriidae, Mesotheriidae) also present some morphological and ontogenetic dental convergences including high-crowned teeth [9,10], and fast dental eruption [11]. These dental innovations, which probably reflect repeated ecological and biological specializations (e.g. specialized herbivory, fast growth; [11]), largely coincide with changing environments and climates starting by the end of the Palaeogene in South America ([12,13], figure 1). However, the relation of notoungulate dental morphology to ecological specializations remains to be explained [11,14,15], and its study should integrate the morphology of the whole masticatory apparatus for a better understanding of their evolution and function. A number of morphological traits of the masticatory apparatus were recently integrated into phylogenetic or ecomorphological analyses of notoungulates [8,16,17]. For instance, Cassini [17] quantitatively investigated the skull shape of Santacrucian notoungulates using geometric morphometrics. However, this study only focused on Miocene genera, and did not consider the early evolution of notoungulates during the Palaeogene, nor the diversity of cranial shape in more derived families (but see [8]). Moreover, it is interesting to know if this diversity, especially cranial convergences, arose in relation to similar external pressures (e.g. large-scale environmental and climatic variations), as suggested for convergent dental crown height increases [11]. None of these studies focused on the origins and evolution of the wide range of shapes (e.g. convergences) of the masticatory apparatus in the entire group, which would contribute to a better understanding of how such a morphological diversity could arise in South America.

Here, we aim to describe the diversity of the masticatory apparatus in notoungulates, from Palaeogene taxa to late-diverging Neogene families by quantifying rostral shape using three-dimensional geometric morphometric analyses. These analyses serve three main objectives: (i) to measure the shape differences between derived notoungulates and early taxa, and assess the main morphological changes involved; (ii) to evaluate the allometric and phylogenetic components which may explain these changes; (iii) to quantify the evolutionary rates of the main morphological changes in order to determine the extent to which they are related to large-scale climatic and environmental variations. This approach will allow an unprecedented characterization of rostrum shape diversity and evolution within a well-documented endemic clade of South American mammals including highly specialized herbivorous forms. More generally, this will permit better understanding of underlying mechanisms at the origin of phenotypic diversification in mammals.

## 2. Material and methods

### 2.1. Material studied

We investigated 70 crania encompassing 26 genera belonging to different clades of notoungulates, and which cover a wide stratigraphic range, from Early Eocene to Pleistocene (figure 1*a*; electronic supplementary material, tables S1 and S2). Specimens are housed in the collections of the Muséum National d'Histoire Naturelle (MNHN, Paris, France), the Museo Argentino de Ciencias Naturales (MACN, Buenos Aires, Argentina), the Museo de La Plata (MLP, La Plata, Argentina), the Museo Regional Provincial Padre M. Jesus Molina (MPM-PV, Rio Gallegos, Argentina), the Universidad Nacional de Patagonia ‘San Juan Bosco' (UNPSJB, Comodoro Rivadavia, Argentina), the Museo Nacional de Historia Natural (MNHN-Bol, La Paz, Bolivia), the Museo de Historia Natural, Universidad Nacional Mayor de San Marcos (MUSM, Lima, Peru), the Museo Nacional de Historia Natural, Vertebrate Paleontology (SGOPV, Santiago, Chile), the American Museum of Natural History (AMNH, New York) and the Yale Peabody Museum of Natural History, Yale University (YPM-PU, New Haven, USA).

### 2.2. Geometric morphometric methods

**Figure 2.**
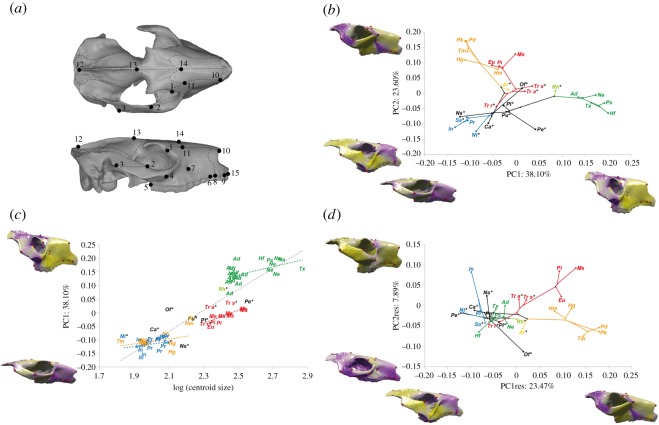
(*a*) Landmarks digitized on the cranium (dorsal view and lateral view) of *Puelia* sp. rendered from photogrammetry and three-dimensional imaging. (*b*) Principal component analyses for crania of notoungulates with phylogenetical relationships and associated virtual deformations with landmarks on the extreme sides of each axis. (*c*) Regression of the first principal component on the centroid size and associated virtual deformations with landmarks. Regression lines are also represented for late-diverging families. (*d*) Principal component analyses for crania of notoungulates with shape data corrected for allometry, phylogenetical relationships and associated virtual deformations on the extreme sides of each axis. Yellow and violet code for increases and decreases in surface area, respectively. For abbreviations figure 1, and ‘asterisks’ characterize Palaeogene forms.

Cranial shape was quantified using 15 anatomical landmarks that mostly cover the rostral part of the skull (figure 2*a*; electronic supplementary material, text S1). This dataset primarily originated from the study of Cassini [17] on Santacrucian South American native ungulates (SANUs, including notoungulates). In order to optimize specimen sampling, some landmarks were removed from the dataset, because we could not place them on partly damaged crania. As a result, only the right side of the cranium was investigated, and if damaged, the left side was used after being mirrored using Geomagic (www.geomagic.com; electronic supplementary material, table S1). When necessary, a few landmarks were also virtually and partially reconstructed, only on partly missing structures of a few specimens, by comparison with other specimens from the same species (electronic supplementary material, table S1). For greater precision, the impact of the most important partial reconstructions was estimated for the cranium of *Campanorco inauguralis* (MLP79-IV-16-1), for which different stages of slight retro-deformations were performed using Cinema4D (https://www.maxon.net/fr/produits/cinema-4d/cinema-4d/; electronic supplementary material, text S2). Morphological distances between the different retro-deformed shapes were calculated using a geometric morphometric analysis (see below) and showed that differences were not significant at the scale of our study (i.e. in comparison with the morphological differences between other taxa; electronic supplementary material, figure S1), which focuses on interspecific variation. Mandibles were not considered in this study because of their scarcity and highly damaged condition in the notoungulate fossil record, especially for Palaeogene taxa.

Digital data of Santacrucian specimens of notoungulates (i.e. *Adinotherium*, *Nesodon*, *Protypotherium*, *Hegetotherium*, *Interatherium* and *Pachyrukhos*; figure 1*a*) were previously acquired using a Microscribe 3D digitizer (*n* = 31) [17]. These data were complemented by digitization of reconstructed meshed skulls using mainly photogrammetry (*n* = 28; using Agisoft PhotoScan, www.agisoft.com) and also surface scanning (*n* = 3; NextEngine 3D scanner), and X-ray microtomography (*n* = 9; platform AST-RX, GE's Phoenix v|tome|x 240 L, MNHN Paris, France; Nikon XTek XT H225 at the Harvard Center for Nanoscale Systems, Cambridge, USA; scanner OPTIMA CT660 from Clinica La Condes, Santiago, Chile). The final processing of the meshes was performed using Geomagic. Landmarks were then digitized using the ‘LANDMARK editor’ (http://graphics.idav.ucdavis.edu/research/EvoMorph). All these methods provide reliable representations of the structures of interest (e.g. [18]), and can be used for quantitative analyses and comparisons of shape variation, involving discrete landmarks.

### 2.3. Statistical analyses

All configurations (sets of landmarks) were superimposed using the Procrustes method of generalized least squares superimposition (GLS scaled, translated and rotated configurations so that the intra-landmark distances are minimized; electronic supplementary material, table S3) following Rohlf [19] and Bookstein [20]. Shape variability of the cranium was visualized by principal components analysis (PCA; figure 2*b*). Phylogenetic relationships were plotted into the morphospace described by the first PCs (mean coordinates for each genus), using the phytools R package ([21]; figure 2*b*). Analysis and visualization of patterns of shape variation were performed with the software package MORPHOTOOLS [22]. The cranium of *Puelia* (MLP 67-II-27-27), which sits in the middle of the resulting morphospace and most closely represents the mean shape, was used in order to create virtual deformations illustrating the observed shape variation.

A multivariate regression of the Procrustes coordinates on the logarithm of the centroid size allowed us to evaluate the effects of allometry on shape by calculating the allometric vector shape (AVS), and residuals of this regression of shape on size (figure 2*c,d*). As a result, PCres corresponds to principal components of a PCA performed on shape data corrected for allometry. Additionally, univariate regressions were undertaken using the log centroid size of each specimen and PC1 (figure 2*c*). Phylogenetic relationships were also plotted on the morphospace described by PCres1 and PCres2 (figure 2*d*).

**Figure 3.**
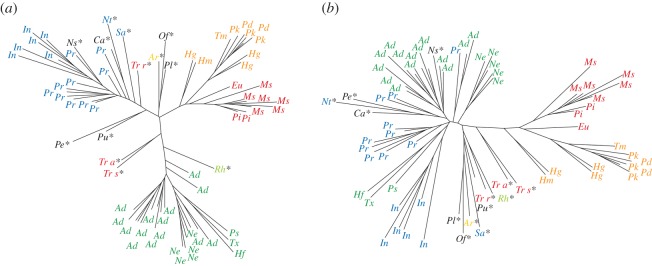
Tree illustrating morphological distances between notoungulate cranial shapes resulting from neighbour-joining analyses on data (*a*) not corrected and (*b*) corrected for allometry. For abbreviations figure 1, and ‘asterisks’ characterize Palaeogene forms.

The subsequent analyses were performed by using the first PCs representing 95% of the shape variance (17 PCs and 16 PCres for shape data uncorrected and corrected for allometry, respectively). We calculated neighbour-joining trees on these data to evaluate the morphological distance between each specimen using the MASS R package ([23]; figure 3). We also calculated a multivariate K-statistic to assess the amount of phylogenetic signal in these data using the geomorph R package [24].

**Figure 4.**
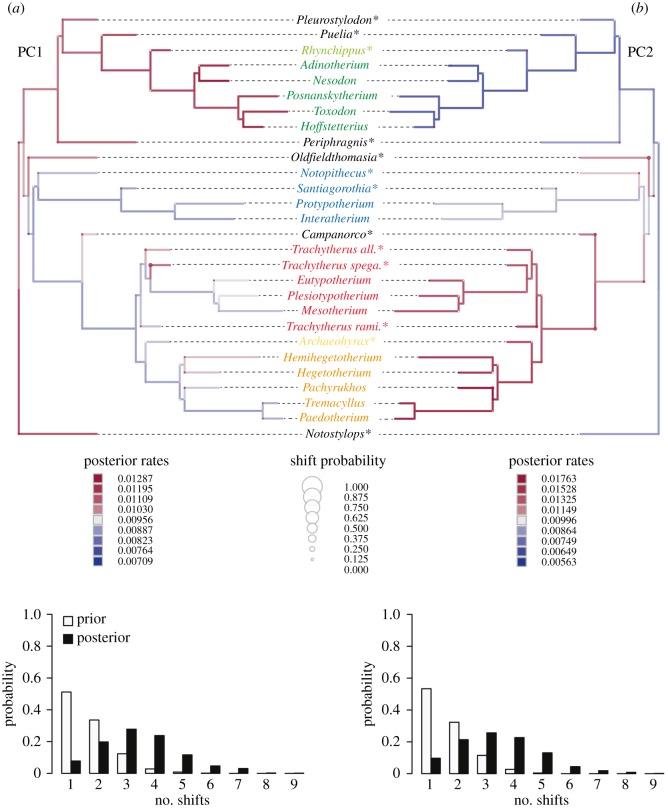
Evolutionary rates associated with cranial shapes in notoungulates, illustrated by the probabilities of the number of shifts on the phylogenetic tree according to (*a*) PC1 and (*b*) PC2. ‘Asterisks’ characterize Palaeogene forms.

We investigated variation in rates of evolution of rostral shape in notoungulates on the two first PC axes (uncorrected for allometry) describing the main morphological variation, and using a relaxed Brownian motion (BM) model with the function *rjmcmc.bm* as implemented in the geiger R package [25]. This flexible model allows the identification of rate changes (shifts) in trait evolution across lineages using a Bayesian method based on reversible jump Markov chain Monte Carlo (MCMC) [26]. We also evaluated the fit of alternative evolutionary models using maximum-likelihood inference for comparison, which is described in electronic supplementary material, text S3. The analyses were performed on the two separate PC axes, which are the most informative, with the notoungulate tree scaled to unit height (figures 1*a* and 4). The tree used corresponds to a composite time-scaled cladogram created in Mesquite v. 3.04 [27] using recent phylogenetic studies [7,16,28], with first and last occurrences of each taxon as references, and for which each divergence was arbitrarily set 1 Myr before the first occurrence of the oldest taxon of the node (electronic supplementary material, table S2). The MCMC was run for 1 × 10^6^ generations sampling every 1000 generations. Two independent runs were used to assess convergence and the first 25% of the chains were discarded as burn-in. The evolutionary rates and measurement error parameters estimated by the model were assigned a weakly informative half-Cauchy prior distribution with scale 25 [29] and the number of shifts was assigned a Poisson distribution with the expected number of shifts set to log(2), which places 50% prior probability on the hypothesis of no shifts. We also implemented a posterior simulation-based analogue of Akaike's information criterion (AIC) through MCMC (AICM; [30]).

## 3. Results

The morphospace depicted by the two first PCs (figures 2*b*; electronic supplementary material, figure S2) accounts for more than 60% of total shape variation. Most taxa from late-diverging families are located at the extremities of this morphospace, whereas most of Palaeogene taxa are located in the centre of the morphospace. Negative values of PC1 (approx. 38%) are characterized by a shallow cranium with an elongated zygomatic arch, and a larger frontal but a shorter snout (figure 2*b*). Conversely, positive values are associated with a higher cranium bearing a large and massive rostrum including an anteriorly protruding premaxilla and a dorsoventrally curved dentition (i.e. with mesial dental landmarks higher than the distal one), whereas the zygomatic arch is short and robust, and the fronto-parietal area is short and located higher than the snout. The hegetotheriids and interatheriids both show negative values on PC1, whereas the toxodontids occupy their own part of morphospace on the positive side of PC1. On PC2 (figure 2*b*), the shape variation (approx. 24%) is mainly associated with the development of an enlarged and oblique zygomatic plate towards positive values. This plate is absent toward negative values, replaced by a slender descending process, prominent in interatheriids. On the positive side, both hegetotheriids and most mesotheriids are characterized by a strongly enlarged zygomatic plate combined with a reduced dentition (i.e. reduced distance between the third molar and the first functional premolar), an enlarged nasal region, an anteriorly reduced premaxilla and a short fronto-parietal region. PC3 explains about 8% of the shape variance (electronic supplementary material, figure S2). On the negative side, the cranium is compressed in its length, width and height, except in the frontal region; this side includes most of the Palaeogene taxa, derived hegetotheriids and derived toxodontids. Conversely, on the positive side, the cranium is much more expanded except in its middle portion; this trait is shown in derived interatheriids (i.e. *Interatherium*) and derived mesotheriids.

Allometry explains 37.6% of the rostral shape variation in the whole dataset, according to the AVS. A regression between centroid size and PC1 (from the PCA uncorrected for allometry) is highly significant (*r*^2^ = 0.80, *p *< 0.001, figure 2*c*). This result is similar to regression between the centroid size and AVS (electronic supplementary material, figure S3). As a result, shape variations expressed on the PC axes calculated using residuals (PCres1 and PCres2) roughly correspond to that of PC2 and PC3, respectively (figure 2*d*; electronic supplementary material, figures S2b and S3b). It is also noteworthy that most late-diverging families of notoungulates follow a slightly different allometric trend from the mean regression, in having a regression line with a lower slope, even if these regressions present a low *r*^2^ (figure 2*c*; electronic supplementary material, table S4). This observation should be considered with caution, because several notoungulates clearly depart from this regression (e.g. *Notopithecus*, *Oldfielthomasia*, *Toxodon*, *Tremacyllus*).

If we take into account most of the variations uncorrected for allometry (95%), the four late-diverging families are clearly delimited according to the neighbour-joining analyses (figure 3). Most Palaeogene taxa are less clearly distinctive—they branch close to the ‘root' of the phenetic tree, even if some of them show characteristic and distinct shapes (e.g. *Notostylops*, *Notopithecus*, *Campanorco* and *Rhynchippus*; figure 3*a*). It is worth noting that the different species of *Trachytherus* are distant from other mesotheriids (i.e. mesotheriines), and positioned closer to toxodontids or interatheriids. When the neighbour-joining analysis is performed on PCres, meaning that the allometric effect is removed, hegetotheriids and mesotheriines are the most strongly delimited taxa (figure 3*b*).

Our analysis of phylogenetic signal shows that shape has a significant and strong phylogenetic signal (*K*_mult_: 1.016, *p *< 0.001; figure 2*b*), and this signal is less important for data corrected for allometry, but still highly significant (*K*_mult_: 0.710, *p *< 0.001; figure 2*d*).

Frequent shifts were identified by the relaxed BM model on the two first PC axes (posterior distribution of shifts differs from the prior distribution, figure 4). However, the shift probability across branches is low (highest supports are approximately 10%, e.g. the branch leading to *T. spegazzinianus* on PC1, the branch leading to *Campanorco* and the branch leading to its sister clade on PC2) indicating that there are no significant shifts but rather a more diffuse change towards slightly higher rates in the clade including the Toxodontidae and early diverging taxa on PC1 (figure 4*a*). On PC2, a small increase in rate is evident in the clade encompassing *Archaeohyrax*, the Hegetotheriiidae and the Mesotheriiidae (figure 4*b*). However, these rate changes are marginal, and we found substantial and better support for a simpler BM model with constant rate of evolution across lineages according to the AICM criterion (ΔAICM PC1 = 19.3, ΔAICM PC2 = 13.5), rather than for the alternative evolutionary models tested, such as the climatic model (see electronic supplementary material, Text S3).

## 4. Discussion

### 4.1. An important allometric component driving rostral shape

Allometry explains the shape of the notoungulate masticatory apparatus to a considerable extent, being responsible for more than one-third of total rostral shape variation. This result corroborates the study of Cassini ([17]; 38.5%) on Santacrucian notoungulates, notwithstanding the addition of many specimens of intermediate sizes filling previous gaps between the small-sized Interatheriidae and Hegetotheriidae, and the large-sized Toxodontidae. This means that size impacts rostral shapes across the entire size range of notoungulates as seen in extant perissodactyls (40%; [17]), and contrary to artiodactyls (7.4%; [17]), which are currently taxonomically much more diverse. Small notoungulate species tend to have a wider but flatter cranium with a reduced snout, while large species have a higher and more robust cranium and zygomatic arches with a large rostrum. These trends are not unique to notoungulates as small mammals are generally shorter faced than large ones [31]. This is also the case for many other vertebrates (e.g. [32]). As previously suggested [31,33,34], allometry can act as a constraint by reducing the range of directions of cranial shape changes. These size-related constraints, which may also vary within the order (e.g. Mesotheriidae), could in fact partly explain the cranial diversity observed in notoungulates, which blossomed during the Oligocene [8,35], with the emergence of new families (e.g. Mesotheriidae, Hegetotheriidae, Toxodontidae). Furthermore, allometry can also accelerate shape change by producing significant morphological differences along lines of least evolutionary resistance [34,36]. In addition, allometric patterns have also been shown to be labile and adaptive, and contribute to evolvability [37,38]. This phenomenon of important size and shape changes seems to be observed right from the beginning of the evolutionary history of notoungulates (i.e. from the Palaeogene).

Mesotheriids and hegetotheriids clearly depart from the allometric pattern in having enlarged zygomatic plates associated with a reduced dentition. This pattern is enhanced in derived species (i.e. mesotheriines, pachyrukhines), which present a more advanced rodent-like morphology including ever-growing incisors (e.g. [3,7]). Moreover, both families maintained a distinctive but still convergent morphology across a considerable range of sizes (between 0.5 and 100 kg [39]). More generally, notoungulates had a wide range of body masses, from 0.5 kg up to 4 tons, which is remarkable regarding their moderate taxonomic diversity (13 families) in comparison with Holarctic ungulates [40]. This considerable size diversity within notoungulates (i.e. from small rodent-like hegetotheriids to large rhino-like toxodontids) undoubtedly indicate niche partitioning. Skull size is partly related to rostral shape within the group, and thus might reflect different ecological specializations (e.g. foraging and processing food). Observations regarding the size diversity and ecological analogy of notoungulates with many extant mammals, including rodents, are substantiated by postcranial remains, which may also reflect cursorial, saltatorial, semi-aquatic or fossorial adaptations [8,41–43]. Size variation might consequently be one of the main factors driving the diversification of notoungulates during their long and mostly endemic evolution in South America.

### 4.2. A strong phylogenetic signal

Phylogenetic signal in morphometric data has long been a matter of discussion [44]. Despite indications that morphometric data are sometimes problematic in reconstructing accurate phylogenies for divergences deeper than a few million years [45], recent investigations demonstrated significant phylogenetic signal for divergences older than 10–20 Ma in various groups of animals [46–48]. Interestingly, Cassini [17] showed that there are strong phylogenetic constraints on the cranial landmark data of different groups of mammals (e.g. the modern artiodactyl and perissodactyl ungulates, hyraxes and macropodids). Our investigation on the rostral shape of notoungulates shows that morphometric data both uncorrected and corrected for allometry are largely congruent with the phylogenetic pattern supported by discrete craniodental characters for Notoungulata [16].

There is an obvious caveat in comparing the phylogenetic signals of two morphological datasets based on the skull (i.e. our morphometric data with the cladistic analysis of Billet [16]) because their data may partly overlap. It is, nevertheless, noteworthy that the morphometric data on the rostrum are also largely congruent with earlier systematic accounts that were mostly based on other anatomical partitions, such as teeth or postcranial material (e.g. [5,49,50]). With respect to these early systematic accounts, rostral shape can be regarded as carrying a strong phylogenetic signal in notoungulates. More specifically, morphometric data uncorrected for allometry provide excellent discrimination of the undisputed four Neogene families of notoungulates. The discrimination of Palaeogene notoungulates is relatively weaker. This would suggest that the phylogenetic signal contained in our morphometric dataset is more concentrated in differences among taxa that exhibit distinct derived shapes accumulated over a long span of time. The future addition of other Palaeogene representatives to our dataset could, however, provide a better resolution of some Palaeogene families (e.g. Notostylopidae, Notohippidae), though many of them may also represent poorly defined paraphyletic entities [16].

The morphometric analyses of rostral shape also clearly indicate strong resemblances between hegetotheriids and mesotheriines, even when the effect of allometry is removed. These similarities principally include the acquisition of a large oblique zygomatic plate, a reduced dentition combined with an enlarged diastema. According to the phylogeny proposed by Billet [16], these rodent-like features were acquired convergently in mesotheriines and hegetotheriids. Though hegetotheriids and mesotheriines are not sister taxa, they are closely related within Typotheria, a suborder of notoungulates [16]. For this reason, their striking convergence evokes the disputed concept of parallelism. Parallelism generally refers to independently derived resemblances resulting from the same underlying genetic changes, a phenomenon which may be more likely to occur over small phylogenetic distances [51] but whose definition and detection are problematic [52]. In any case, the independent evolution of a rodent-like masticatory apparatus in mesotheriids and hegetotheriids represents an impressive series of convergences between close relatives, independent of size, and which may find its roots in similar functional specializations of their masticatory apparatus.

Finally, our analyses also highlight a large morphological gap between early diverging mesotheriids (i.e. ‘trachytheriines’) and late-diverging ones (mesotheriines), a pattern which is not found for the other Neogene families: Toxodontidae, Hegetotheriidae and Interatheriidae. This gap suggests that rostrum morphology underwent substantial changes within the mesotheriid family. The analyses of the evolutionary rates of PC1–2 along the notoungulate tree show, however, no support for a significant shift at the node separating the two subfamilies. These analyses also corroborate the result of the *K*_mult_ (approx. 1.0) meaning that rostral shape in notoungulates evolved under BM rather than showing shifts towards adaptive optima [24].

### 4.3. No clear influences of large-scale environmental and climatic variations on notoungulate rostral shape diversification

Our analyses of evolutionary rates demonstrate that the major rostral shape changes probably followed diffuse and rather weak variations of evolutionary rates (i.e. almost BM) across the entire notoungulate tree. Nonetheless, accelerations of morphological evolution, though weak, were observed in toxodontids in concert with size and associated rostral robustness increases, and were also noted in mesotheriids and hegetotheriids in association with the acquisition of a rodent-like masticatory apparatus. The convergent cranial changes observed in rodent-like notoungulates, as well as increasing body size in some groups, might be partly associated with slowly changing environments from the end of the Eocene to the beginning of the Miocene ([53], figure 1*c*). It is interesting to note that the rodent-like rostral shape is reminiscent of some sciuromorphous rodents, such as groundhogs or beavers. Rodent-like taxa have strongly developed zygomatic plates allowing the insertion of the anterior masseter muscles [54–56]. The rostral morphology of rodent-like notoungulates may have improved the bite force at the incisor level, as demonstrated in rodents [54,57], and could be related to fossorial or ‘woodpecker' habits (i.e. such as striped possum and aye-aye [17,41,42,58]). As a result, this shape may reflect local functional adaptations.

More generally, according to the apparent ‘constant' rate of rostral shape evolution in notoungulates, no clear link to large-scale environmental and climatic variations (i.e. increasing aridity, episodes of intensified Andean uplift) occurring in South America from the Middle Eocene to the Middle Miocene [12,13,53] can be suggested (see also electronic supplementary material, Text S3), pending further ecological analyses. This result on rostral shape contrasts with the signal provided by multiple acquisitions of ever-growing dentitions in late-diverging families, which coincide well with cooling and intense volcanism events slowly generating increasing aridity at mid-Cenozoic periods [11,14,53]. This means that external selective forces or response to these forces may have been very different between crown height and rostral shape. Nevertheless, we cannot discard that, due to limitations in the fossil record, the reduced sampling of Eocene notoungulates in our study (e.g. no Typotheria sampled for the Late Eocene; figure 1) may have hampered the detection of an evolutionary rate shift, most particularly around key periods such as the Late Eocene–Early Oligocene. In addition, the selective pressure potentially exerted by large-scale environmental variations on the evolution of crown height in notoungulates remain to be tested statistically to validate the hypothesis of differential levels of selective forces on the various components of the masticatory apparatus. If it is indeed the case, this would partly explain the mosaic evolution of this morpho-functional complex in notoungulates, which could reflect their putative ecological diversification [3]. This mosaic evolution also suggests that notoungulates accommodated differently the dental height increase in the structure and function of their masticatory apparatus.

For instance, Mesotheriidae and Hegetotheriidae convergently display a rodent-like masticatory apparatus characterized in derived species by a reduced number of teeth and a large diastema between ever-growing cheek teeth and incisors, but their dental morphology differs (i.e. distinct occlusal shape and relief). It should also be noted that, in the course of rodent evolution, different rostral shapes allowing improvement of incisor efficiency, such as sciuromorphy, occurred many times and approximately simultaneously according to Wood [59]. Such a diversity of rostral shape in rodents was primarily considered as non-selectively emerging [60,61], even if the different masticatory types are defined as biomechanically different in extant species [57], meaning that some morphotypes were then probably positively selected. Similar cases of non-selective emergence and evolution might be hypothesized considering notoungulate rostral shape, in relation to ever-growing dentition. However, directional selection probably occurred, considering *inter alia* some cases of convergent evolution (e.g. size increase, rodent-like shape). A process where species-specific habitats, which fluctuate in a randomly changing environment might also explain the reasonable fit provided by the BM model in our study (e.g. [62]). Consequently, hypotheses of specific adaptations of notoungulates to their fluctuating habitats can also be suggested, given that previous studies assumed browsing to grazing habits for late-diverging notoungulates in order to explain the diversity of both cranial and dental shapes [14,41,42,63,64]. Although inferences are relevant to some extent when considering extant morphological analogues, the details of their morpho-functional feeding ecology still need to be more accurately defined owing to the complexity introduced by diverging rostral shape (except rodent-like) and converging dental crown height in these taxa.

In sum, if the diversification of cranial shapes appeared early during the course of notoungulate evolution (i.e. since the Eocene, with *Notostylops*, *Notopithecus* and *Campanorco*), the pace of this morphological diversification was maintained through most of the Cenozoic. This diversification was closely related with body size evolution, while influences of large-scale environmental variations remain unclear. It is interesting to note that a preliminary analysis suggested that the disparate evolution of body sizes experienced by notoungulates did not show robust relationships with global cooling and environmental variations in South America [11]. Environmental and climatic modifications, as well as geological events occurring between 45 and 20 Ma might nonetheless have contributed to the appearance of new ecological niches for notoungulates, contributing to their diversification. Our results emphasize the necessity of focusing on the different components shaping separate units of the masticatory apparatus in order to better understand the diversification of cranial shape changes in mammals with respect to environmental changes and ecological adaptation.

## Supplementary Material

Fig. S1

## Supplementary Material

Fig. S2

## Supplementary Material

Fig. S3

## Supplementary Material

Table S1

## Supplementary Material

Table S2

## Supplementary Material

Table S3

## Supplementary Material

Table S4

## Supplementary Material

Text S1

## Supplementary Material

Text S2

## Supplementary Material

Text S3

## Data accessibility

Supporting data are accessible in electronic supplementary material, table S3.
