# Supplementary figures and images for: Differential influences of allometry, phylogeny and environment on the rostral shape diversity of extinct South American notoungulates
Source: R Soc Open Sci. 2018 Jan 31;5(1):171816. doi: 10.1098/rsos.171816 (PMC5792951; doi:10.1098/rsos.171816)

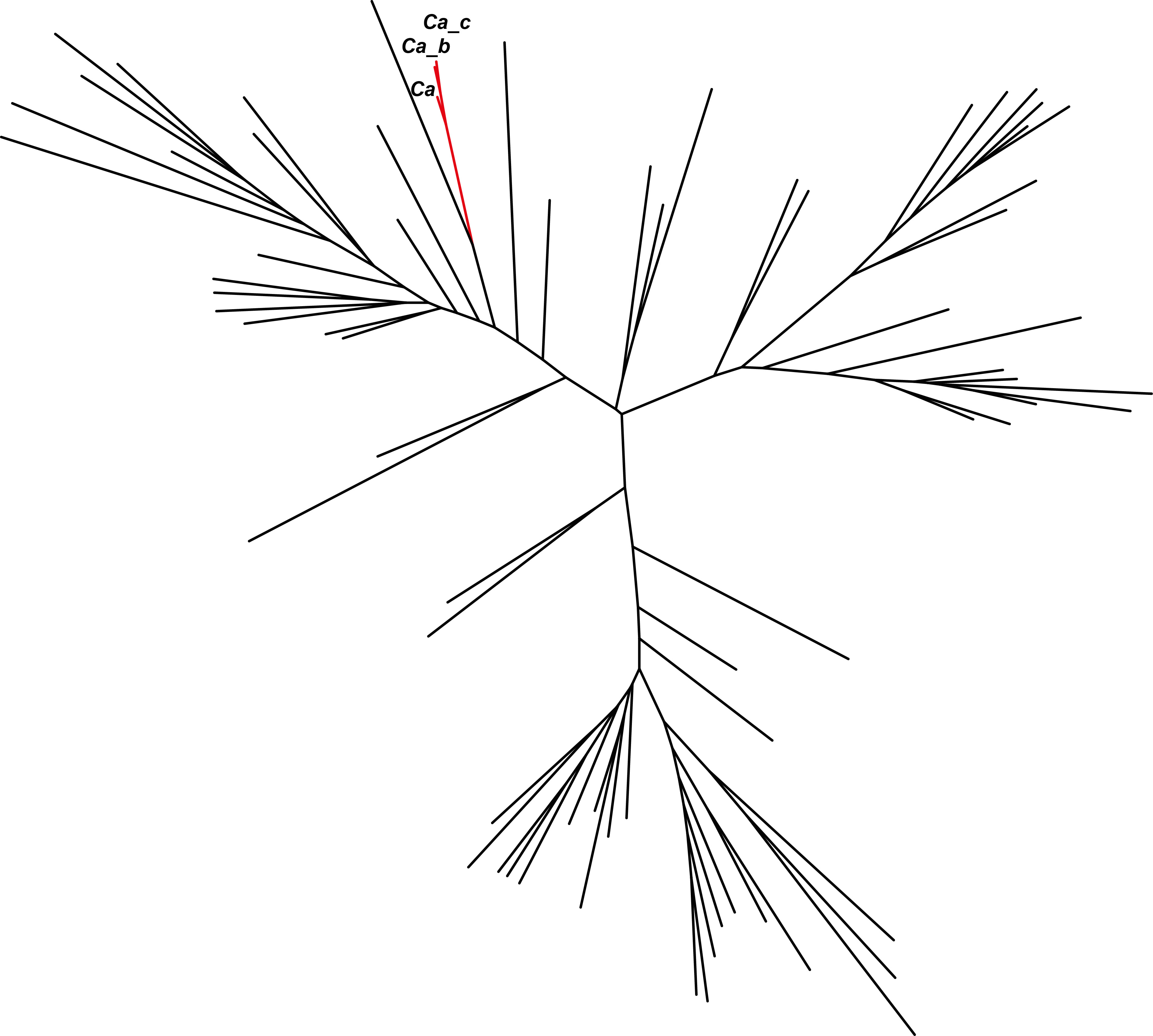

Supplement: Fig. S1 [file rsos171816supp1.jpg]

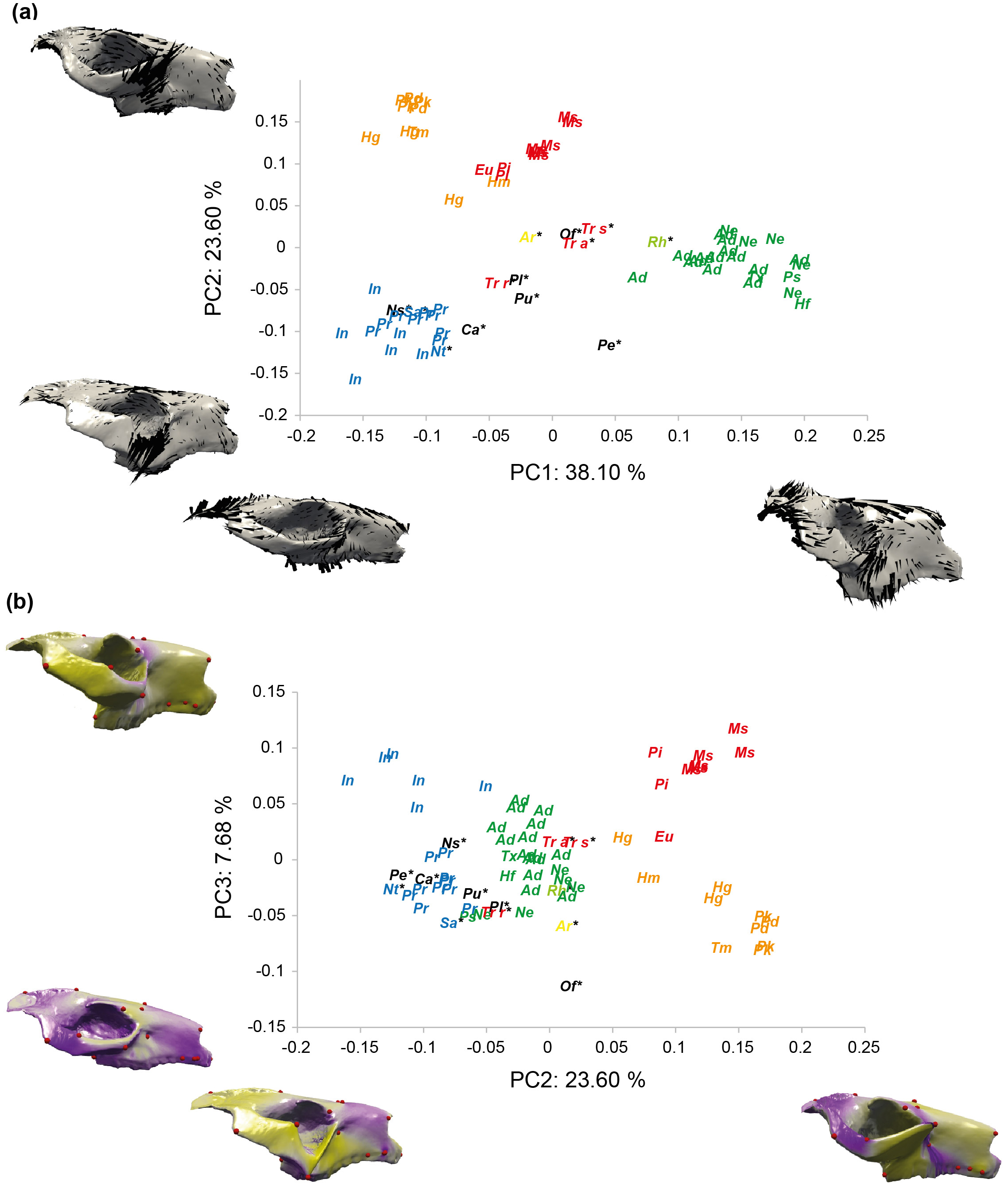

Supplement: Fig. S2 [file rsos171816supp2.jpg]

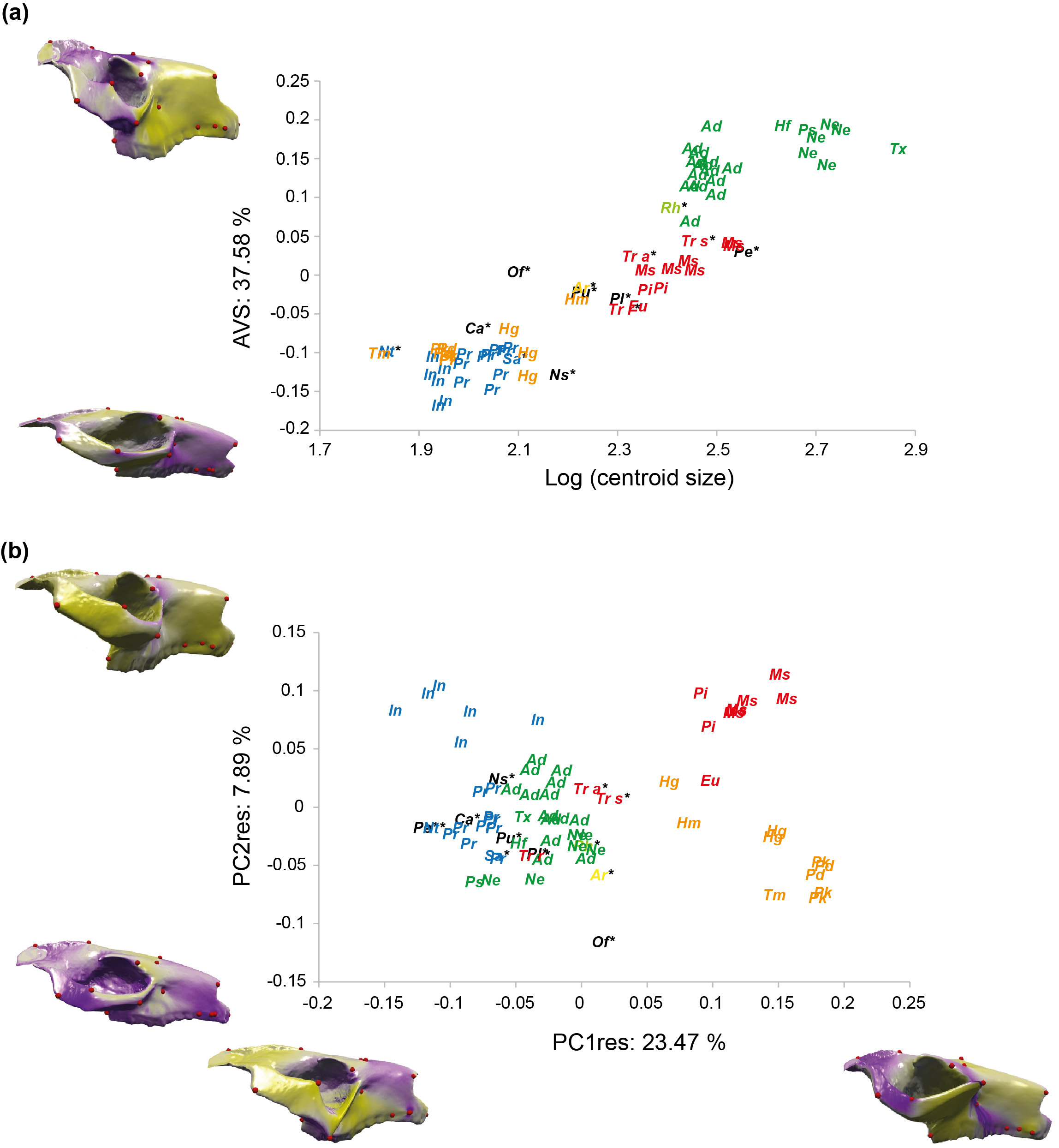

Supplement: Fig. S3 [file rsos171816supp3.jpg]
