# Supplementary material for: Differential influences of allometry, phylogeny and environment on the rostral shape diversity of extinct South American notoungulates
Source: R Soc Open Sci. 2018 Jan 31;5(1):171816. doi: 10.1098/rsos.171816 (PMC5792951; doi:10.1098/rsos.171816)
Supplement: Text S1 [file rsos171816supp8.docx]

**Text S1** List of cranial landmarks

Landmarks 1: Orbital margin of the fronto-lacrimal suture

Landmarks 2: Jugo-squamosal suture on the dorsal margin of the zygomatic arch

Landmarks 3: Jugo-squamosal suture on the ventral margin of the zygomatic arch

Landmarks 4: Anterior most origin of the masseter muscle

Landmarks 5: Distal alveolar margin of the third molar

Landmarks 6: Mesial alveolar margin of the first functional premolar

Landmarks 7: Lower margin of the infraorbital foramen

Landmarks 8: Intralveolar margin of the premaxillary-maxillary suture

Landmarks 9: Distal alveolar margin of the lateral most incisor

Landmarks 10: Premaxillary-nasal suture at the margin of the nasal aperture

Landmarks 11: Junction between the nasal, frontal and maxilla

Landmarks 12: Occipito-parietal suture

Landmarks 13: Fronto-parietal suture

Landmarks 14: Fronto-nasal suture on the sagittal plane

Landmarks 15: Premaxillary suture at the alveolar margin
