# Supplementary material for: Differential influences of allometry, phylogeny and environment on the rostral shape diversity of extinct South American notoungulates
Source: R Soc Open Sci. 2018 Jan 31;5(1):171816. doi: 10.1098/rsos.171816 (PMC5792951; doi:10.1098/rsos.171816)
Supplement: Text S2 [file rsos171816supp9.docx]

**Text S2** Details concerning the different retro-deformation stages of *Campanorco inauguralis* MLP79-IV-16-1 (see figure)

- Step 1: 35% tilt toward the left side of the specimen

- Step 2: 8% tilt (translation) toward the anterior left side of the specimen

Result of steps 1+2 -> **Ca_b**

- Step 3: 15% tilt (translation) toward the upper left side of the specimen

Result of steps 1+2+3 -> **Ca_c**

- Step 4: 3 mm enlargement of 45% of the upper part of the cranium (i.e. above the naso-maxillary suture + fronto-parietal bones)

Result of steps 1+2+3+4 -> **Ca**

**
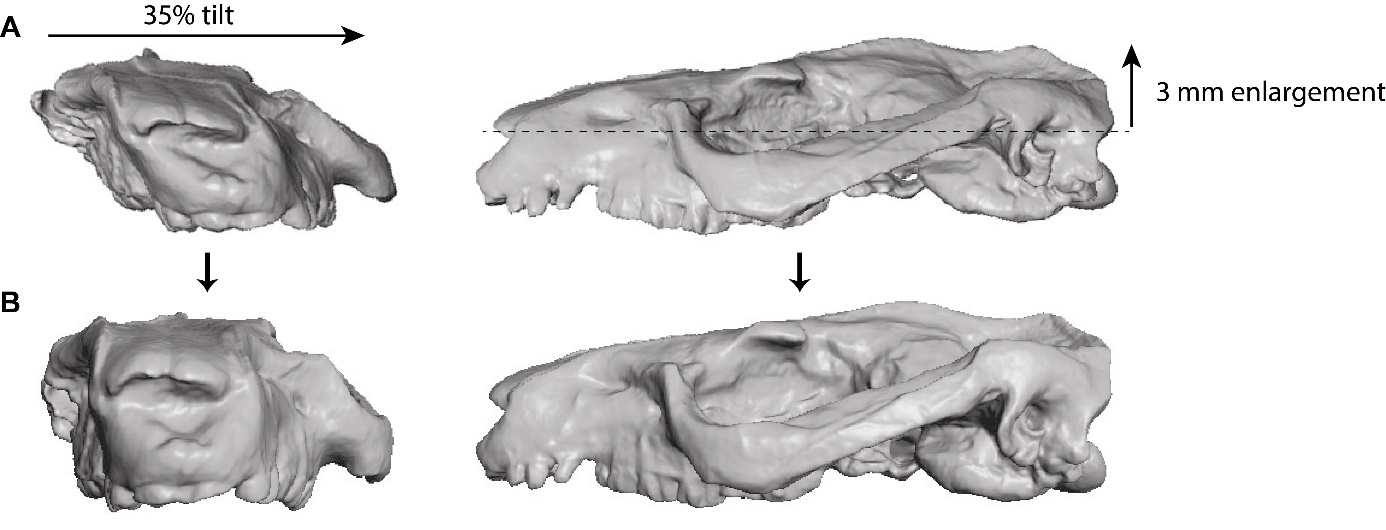
**

Figure showing the main retro-deformation steps between **A.** the original skull and **B.** the final model of *C. inauguralis* (after all the steps: **Ca**)
