# Supplementary material for: Differential influences of allometry, phylogeny and environment on the rostral shape diversity of extinct South American notoungulates
Source: R Soc Open Sci. 2018 Jan 31;5(1):171816. doi: 10.1098/rsos.171816 (PMC5792951; doi:10.1098/rsos.171816)
Supplement: Text S3 [file rsos171816supp10.docx]

**Text S3**

**Supplementary material and methods:**

In addition to the relaxed-BM model fit using Bayesian mcmc on PC1 and PC2 (see main text), we compared the relative fit of seven alternative models of traits evolution by maximum likelihood (ML) inference. We considered the classical Brownian motion (BM) and an Ornstein-Uhlenbeck (OU) process with a single (stationary) optimum (Felsenstein 1985; Hansen 1997). We also considered parameterizations of the BM process that involve a trend in the ancestral trait value (BM-trend), as well as a trend from the ancestral trait value at the root of the tree to the primary optimum for the Ornstein-Uhlenbeck process (OU-trend) that are uniquely identifiable on fossil (non-ultrametric) trees. These models assume constant dynamics of trait evolution but a directional drift of the clade average value that might be interpreted as shifts in evolutionary rates in some models. Finally, we fitted time-dependents models of trait evolution where the evolutionary rates are allowed to change over time: the Early-burst/Acceleration (EB/AC) model where the evolutionary rate can increase or decrease over time (Blomberg et al. 2003; Harmon et al. 2010), and climatic models where the rate can changes as an exponential (ClimExp) or linear (ClimLin) function of climate variation through time (Clavel and Morlon 2017). For the climatic models we used the Cenozoic temperature curve reconstructed by Cramer et al. (2011) to assess for an effect of global changes on notoungulate evolution. All models fit were performed on the R statistical environment using the mvMORPH (Clavel et al. 2015) and RPANDA (Morlon et al. 2016) packages.

**Supplementary results:**

For rostral shape evolution on PC1, model comparison (Table S4.1) using the AIC criterion (AICc) shows that BM has the best support (lowest AICc score/highest AIC weight) followed by the EB/AC and ClimLin models. These two latter models suggest an overall slight decrease in evolutionary rates through time, as also suggested by the Bayesian analysis (Figure 4a). A non-parametric LRT (log-likelihood ratio) test further shows that the BM fit is not significantly different from the EB/AC model (p-value>0.05). For rostral shape variations on PC2, model comparison (Table S4.2) shows that the BM-trend model has a significant support over the simpler BM model (ΔAICc > 2 – see Burnham and Anderson 2002). The trend depicted by the BM-trend model mainly represents the changes from early-divergent taxa to the Mesotheriidae and Hegetotheriidae (see Figure 2 in the main text). Such a trend is in accordance with the Bayesian analysis using the relaxed BM model (Figure 4b), for which slightly higher rates – although marginally supported – were noticed in these clades. In any case, the more parameterized climatic, EB and OU models are the least well supported. Further analysis with more complete taxon sampling will be required to obtain fine-grained analysis of rostral shape evolutionary dynamic in the Notoungulates while increasing statistical power.

**Table S3.1.** Model comparison on PC1. AICc is the second order (small sample size) Akaike Information Criterion ; ΔAICc is the difference in AICc score with the best fit model; AICw is the Akaike weight which represent the relative model support over the set of considered models - Burnham and Anderson (2002). See text for models descriptions.

| Model | Rank | AICc | ΔAICc | AICw |
| --- | --- | --- | --- | --- |
| BM | 1 | -82.9 | 0.000 | 0.32069 |
| EB/AC | 2 | -82.1 | 0.811 | 0.21377 |
| ClimLin | 3 | -81.9 | 1.007 | 0.19383 |
| BM-trend | 4 | -81.3 | 1.650 | 0.14057 |
| ClimExp | 5 | -80.9 | 2.035 | 0.11595 |
| OU | 6 | -76.1 | 6.761 | 0.01091 |
| OU-trend | 7 | -74.3 | 8.631 | 0.00429 |

**Table S3.2.** Model comparison on PC2. Legend same as in Table S4.1.

| Model | Rank | AICc | ΔAICc | AICw |
| --- | --- | --- | --- | --- |
| BM-trend | 1 | -82.3 | 0.00 | 0.64126 |
| BM | 2 | -79.7 | 2.60 | 0.17505 |
| EB/AC | 3 | -77.7 | 4.64 | 0.06297 |
| ClimExp | 4 | -77.2 | 5.10 | 0.05018 |
| ClimLin | 5 | -76.4 | 5.90 | 0.03356 |
| OU-trend | 6 | -76.0 | 6.33 | 0.02704 |
| OU | 7 | -74.0 | 8.33 | 0.00994 |

**Supplementary References:**

Blomberg S.P., Garland T.J., Ives A.R. 2003. Testing for phylogenetic signal in comparative data: behavioral traits are more labile. Evolution. 57:717–745.

Burnham K.P., Anderson D.R. 2002. Model selection and multi-model inference: a practical information-theoric approach. New York: Springer-Verlag.

Clavel J., Escarguel G., Merceron G. 2015. mvmorph: an r package for fitting multivariate evolutionary models to morphometric data. Methods Ecol. Evol. 6:1311–1319.

Clavel J., Morlon H. 2017. Accelerated body size evolution during cold climatic periods in the Cenozoic. Proc. Natl. Acad. Sci. 114:4183–4188.

Cramer B.S., Miller K.G., Barrett P.J., Wright J.D. 2011. Late Cretaceous-Neogene trends in deep ocean temperature and continental ice volume: reconsiling records of benthic foraminiferal geochemistry (d18O and Mg/Ca) with sea level history. J. Geophys. Res. 116:1–23.

Felsenstein J. 1985. Phylogenies and the comparative method. Am. Nat. 125:1–15.

Hansen T.F. 1997. Stabilizing selection and the comparative analysis of adaptation. Evolution. 51:1341–1351.

Harmon L.J., Losos J.B., Davies J.T., Gillespie R.G., Gittleman J.L., Jennings B.W., Kozak K.H., McPeek M.A., Moreno-Roark F., Near T.J., Purvis A., Ricklefs R.E., Schluter D., Schulte II J.A., Seehausen O., Sidlauskas B.L., Torres-Carvajal O., Weir J.T., Mooers A.Ø. 2010. Early bursts of body size and shape evolution are rare in comparative data. Evolution. 64:2385–2396.

Morlon H., Lewitus E., Condamine F.L., Manceau M., Clavel J., Drury J. 2016. RPANDA: an R package for macroevolutionary analyses on phylogenetic trees. Methods Ecol. Evol. 7:589–597.
